# Global metabolomic alterations associated with endocrine-disrupting chemicals among pregnant individuals and newborns

**DOI:** 10.1007/s11306-024-02219-7

**Published:** 2025-01-25

**Authors:** Jagadeesh Puvvula, Lucie C. Song, Klaudia J. Zalewska, Ariel Alexander, Kathrine E. Manz, Joseph M. Braun, Kurt D. Pennell, Emily A. DeFranco, Shuk-Mei Ho, Yuet-Kin Leung, Shouxiong Huang, Ann M. Vuong, Stephani S. Kim, Zana Percy, Priyanka Bhashyam, Raymund Lee, Dean P. Jones, Vilinh Tran, Dasom V. Kim, Antonia M. Calafat, Julianne C. Botelho, Aimin Chen

**Affiliations:** 1 Department of Biostatistics, Epidemiology and Informatics, Perelman School of Medicine, University of Pennsylvania, Philadelphia, PA USA; 2 College of Arts & Sciences, University of Pennsylvania, Philadelphia, PA USA; 3 College of Nursing, University of Pennsylvania, Philadelphia, PA USA; 4 Carleton College, Northfield, MN USA; 5 Department of Environmental Health Sciences, School of Public Health, University of Michigan, Ann Arbor, MI USA; 6 Department of Epidemiology, Brown University, Providence, RI USA; 7 School of Engineering, Brown University, Providence, RI USA; 8 Department of Obstetrics and Gynecology, College of Medicine, University of Kentucky, Lexington, KY USA; 9 Department of Pharmacology and Toxicology, College of Medicine, University of Arkansas for Medical Sciences, Little Rock, AR USA; 10 Pathogen-Host Interaction Program, Texas Biomedical Research Institute, San Antonio, TX USA; 11 Department of Epidemiology and Biostatistics, School of Public Health, University of Nevada Las Vegas, Las Vegas, NV USA; 12 Health Research, Battelle Memorial Institute, Columbus, OH USA; 13 Department of Environmental & Public Health Sciences, College of Medicine, University of Cincinnati, Cincinnati, OH USA; 14 Division of Pulmonary, Allergy, Critical Care and Sleep Medicine, Emory University, Atlanta, GA USA; 15 Immunology Program, Memorial Sloan Kettering Cancer Center, New York, NY USA; 16 National Center for Environmental Health, U.S. Centers for Disease Control and Prevention, Atlanta, GA USA

**Keywords:** Phenol, Phthalate, Pregnancy, Fetus, Metabolome, Untargeted metabolomics

## Abstract

**Background:**

Gestational exposure to non-persistent endocrine-disrupting chemicals (EDCs) may be associated with adverse pregnancy outcomes. While many EDCs affect the endocrine system, their effects on endocrine-related metabolic pathways remain unclear. This study aims to explore the global metabolome changes associated with EDC biomarkers at delivery.

**Methods:**

This study included 75 pregnant individuals who delivered at the University of Cincinnati Hospital from 2014 to 2017. We measured maternal urinary biomarkers of paraben/phenol (12), phthalate (13), and phthalate replacements (4) from the samples collected during the delivery visit. Global serum metabolome profiles were analyzed from maternal blood (*n* = 72) and newborn (*n* = 63) cord blood samples collected at delivery. Fifteen of the 29 urinary biomarkers were excluded due to low detection frequency or potential exposures during hospital stay. We assessed metabolome-wide associations between 14 maternal urinary biomarkers and maternal/newborn metabolome profiles. Additionally, performed enrichment analysis to identify potential alterations in metabolic pathways.

**Results:**

We observed metabolome-wide associations between maternal urinary concentrations of phthalate metabolites (mono-isobutyl phthalate), phthalate replacements (mono-2-ethyl-5-carboxypentyl terephthalate, mono-2-ethyl-5-hydroxyhexyl terephthalate) and phenols (bisphenol-A, bisphenol-S) and maternal serum metabolome, using q-value < 0.2 as a threshold. Additionally, associations of phthalate metabolites (mono-n-butyl phthalate, monobenzyl phthalate) and phenols (2,5-dichlorophenol, BPA) with the newborn metabolome were noted. Enrichment analyses revealed associations (p-gamma < 0.05) with amino acid, carbohydrate, lipid, glycan, vitamin, and other cofactor metabolism pathways.

**Conclusion:**

Maternal paraben, phenol, phthalate, and phthalate replacement biomarker concentrations at delivery were associated with maternal and newborn serum global metabolome.

**Supplementary Information:**

The online version contains supplementary material available at 10.1007/s11306-024-02219-7.

## Introduction

Endocrine-disrupting chemicals (EDCs) are exogenous compounds that mimic natural hormones (AAPCEH, 2019; Schug & Birnbaum, 2020). Numerous preclinical and observational studies have indicated associations between gestational EDC exposures and adverse newborn outcomes (Diamanti-Kandarakis et al., 2009; Mnif et al., 2011). Although exposure to EDCs has a substantial impact during gametogenesis and the early stage of fetal development, certain adverse outcomes as a result of fetal exposure to EDCs may not appear until adulthood (Mnif et al., 2011). Due to the heightened susceptibility to EDC exposures or environmental chemicals in general, several researchers have proposed biomonitoring across life-stage quantifying the exposome (exogenous and endogenous compounds) to understand underlying mechanisms (Barr et al., 2005; Vrijheid et al., 2014; Marín-Sáez et al., 2024). At a molecular level, EDCs primarily interfered with peroxisome proliferator-activated receptor (PPAR), steroid (estrogen, androgen), and thyroid receptors (Metcalfe et al., 2022; Płotka-Wasylka et al., 2023; Soop et al., 2023). The effect of EDCs extends beyond agonist or antagonist roles in endocrine pathways to include receptor expression, signal transduction, and hormone synthesis/transport/distribution/clearance (La Merrill et al., 2020). This interference can potentially disrupt the endocrine and/or metabolic pathways, consequently altering normal physiology (Alonso-Magdalena et al., 2010; Schug & Birnbaum, 2020).

Most of these receptors affected play roles in metabolic pathways. The PPAR nuclear receptors, generally expressed in adipocytes, hepatocytes, muscle, and endothelial cells, play a key role in the metabolism of fatty acids, carbohydrates, and lipids (Ghassabian et al., 2022; Grygiel-Górniak, 2014). Estrogen receptors, abundantly expressed in the central nervous system, cardiovascular system, ovary, lung, bladder, peripheral/adipose tissues, skeletal muscle, immune cells, hematopoietic cells, and fetal brown adipose tissue, were linked to carbohydrate metabolism (glycolysis, gluconeogenesis) and citrate cycle pathways (Chen et al., 2009; Clegg et al., 2017). Altered thyroid hormone concentrations can influence carbohydrate (gluconeogenesis) and lipid metabolism (Cicatiello et al., 2018). Alterations of these metabolic pathways were generally linked with cellular processes (e.g., fetal development), insulin resistance, adiposity, and cardiometabolic conditions (Padmanabhan et al., 2021).

Human exposure to EDCs can occur through food packaging, personal care products, pharmaceuticals, and medical tubing (Gore et al., 2014; Haggerty et al., 2021). While certain EDCs such as parabens, phenols, and phthalates have a relatively short elimination half-life (less than 24 h), research suggests these EDCs can cross the placental barrier (Hoppin et al., 2002; Mose et al., 2007; Pollack et al., 2016; Rager et al., 2020; Søeborg et al., 2014). Exposure to EDCs, especially during a critical developmental phase such as pregnancy, could have a substantial impact on the peri- or post-natal cardiometabolic programming (Heindel et al., 2017; Philips et al., 2017).

Several human observational studies have focused on biomonitoring non-persistent EDCs, exploring their associations with clinical endpoints in the context of gestational exposures and maternal/newborn phenotypes (Arbuckle et al., 2014; Buckley et al., 2022; Haggerty et al., 2021; Welch et al., 2022). Additionally, a multi-omics study that considered metabolome (177 serum metabolites and 44 urinary metabolites), proteome, and methylome profiles suggested associations between maternal exposures to non-persistent EDCs and omics layers that are linked with phenotypes such as fetal growth, insulin resistance, obesity, metabolic and neuroendocrine disorders (Fabbri et al., 2023). Parenti et al. documented serum/placenta metabolome (using < 62 metabolites detected based on internal standards) changes associated with gestational phthalate exposures, indicating alterations in energy, amino acid, and lipid metabolism (Parenti et al., 2022). Additionally, Thomson et al. comprehensively assessed associations between gestational phthalate exposures and metabolome (maternal, cord, plasma from child at year 1) by restricting metabolites to central carbon metabolism and suggested that gestational exposure to di(2-ethylhexyl) phthalate (DEHP) is linked to the energy metabolism (Thomson et al., 2023). However, there is a gap in addressing potential global metabolomic changes underlying exposure-phenotype associations. This study aims to explore associations between urinary concentrations of 29 exposure biomarkers of non-persistent EDCs (phthalates, phthalate replacements, and phenols) and the global maternal/cord serum metabolome at delivery.

## Methods

### Study participants and sample collection

Between August 2014 and September 2017, 75 pregnant individuals enrolled from the labor and delivery unit at the University of Cincinnati Medical Center, Cincinnati, Ohio. The research protocol was reviewed and approved by the Institutional Review Board at the University of Cincinnati. Eligible participants include individuals aged between 18 and 45 years with singleton pregnancies who were admitted for childbirth and provided written consent upon enrollment. Individuals with a medical history of diabetes, thyroid disorders, cardiovascular, renal, hepatic conditions, cancers impacting pregnancy, or severe maternal or fetal complications at the time of recruitment were excluded from this study.

During the hospitalization for birth, we collected 30 mL of maternal spot urine and 15 mL of maternal non-fasting venous blood. Additionally, we collected 10 mL samples of cord blood (hereafter referred to as newborn) after delivery. Blood samples were collected in BD Vacutainer™ serum separation tubes and allowed to clot for at least 30 min. Then, the samples were centrifuged to separate from blood cells. We then transferred up to 2 mL of serum (maternal/cord) into cryovials. Maternal urine samples were collected using a sterile urine collection cup. Samples were mixed by swirling and aliquoted into 10 mL cryovials. These biospecimens were preserved at -80 °C and shipped to laboratories on dry ice overnight, remaining frozen until analysis. We obtained maternal demographic information, medical history, tobacco use during pregnancy, pre-pregnancy body mass index (BMI), and pregnancy outcomes of our study participants through a questionnaire and by reviewing their medical records.

### Quantifying EDC biomarkers

We measured urinary concentrations of 29 biomarkers (sum of unconjugated and conjugated species) of parabens, phenols, phthalates, and phthalate replacements using on-line solid-phase extraction coupled with high-performance liquid chromatography isotope dilution tandem mass spectrometry (Silva et al., 2007; Ye et al., 2005, 2006). The precision of the phthalate metabolite quantification was calculated as the coefficient of variation (CV); the intraday CV ranged from 1.8 to 13.8%, and the inter-day (3-month window) CV ranged from 2.7 to 14% (Silva et al., 2007). Phthalate metabolite quantification accuracy was measured using synthetic urine spiked with known concentrations (2 distinct levels per metabolite) that showed a good agreement between the theoretical and estimated concentrations (Silva et al., 2007). For phenols, the inter and intraday CV ranged between 6 and 25% (Ye et al., 2005). The accuracy of phenols across four distinct concentrations showed similar concentrations between the theoretical and estimated concentrations (Ye et al., 2005). The intra and inter-day CV for parabens ranged from 5.3 to 10.6% and with an accuracy of 90–110% (Ye et al., 2006). These biomarker urinary concentrations were quantified at the Centers for Disease Control and Prevention (CDC), Atlanta, GA. The involvement of the CDC laboratory did not constitute engagement in human subjects’ research.

Table 1 outlines the limit of detection (LOD) for each chemical biomarker. For biomarkers detected in at least 60% of study participants, we imputed the concentrations reported as below LOD with LOD divided by the square root of two (Lubin et al., 2004). Among the 29 chemical biomarkers assessed from maternal urine, we excluded 15 for further analysis. Notably, because we cannot rule out that exposure to certain EDCs resulted from medical-related procedures during delivery, we excluded four phthalate metabolites (mono-2-ethyl-5-carboxypentyl phthalate, mono-2-ethyl-5-hydroxyhexyl phthalate, mono-2-ethyl-5-oxohexyl phthalate, and mono-2-ethylhexyl phthalate), and three parabens (ethyl paraben, methyl paraben, propyl paraben) (Vandentorren et al., 2011; Yan et al., 2009). We excluded eight biomarkers detected in fewer than 60% of the participants, as outlined in Table 1. Additionally, we obtained the median urinary biomarker concentrations adjusted for urine creatinine from the National Health and Nutrition Examination Survey (NHANES) cycle 2015–2016 and biomarker concentrations among pregnant individuals included in the NHANES cycle 2013–2016 for comparison (CDC).

**Table 1:** Summary statistics of maternal urinary biomarkers (*n* = 72)

Parent compound/group		Biomarker	LOD (µg/L)	% < LOD	Median (IQR)*	2015–2016 NHANES median^*^	Pregnant population NHANES^+^
Low molecular weight phthalates	Diethyl phthalate (DEP)	Monoethyl phthalate (MEP)	1.2	0	13.1 (4.9–47.2)	27.7	41.20 (16.1-110.6)
	Di-n-butyl phthalate (DBP or DnBP)	Mono-n-butyl phthalate (MBP)	0.4	5.3	3.8 (1.9–12.8)	9.9	11.7 (5.7–22.8)
	Di-iso-butyl phthalate (DiBP)	Mono-isobutyl phthalate (MiBP)	0.8	21.3	3.0 (0.9–11.5)	8.3	10.2 (4.4–19.7)
High molecular weight phthalates	Benzylbutyl phthalate (BBzP)	Monobenzyl phthalate (MBzP)	0.3	8.0	1.7 (0.9–7.6)	4.2	5.2 (2.1–13.7)
	Di-n-octyl phthalate (DnOP)	Mono-3-carboxypropyl phthalate (MCPP)^a^	0.4	41.3	0.6 (< LOD-1.8)	1.1	10.7 (5.4–20.9)
	Di(2-ethylhexyl) phthalate (DEHP)	Mono-2-ethyl-5-carboxypentyl phthalate (MECPP)^b^	0.4	0	112 (43.3-269.5)	8.5	NA
		Mono-2-ethyl-5-hydroxyhexyl phthalate (MEHHP)^b^	0.4	0	69.7 (26.6–198.0)	5.5	6.7 (3.1–13.1)
		Mono-2-ethyl-5-oxohexyl phthalate (MEOHP)^b^	0.2	0	61.0 (16.35–160.0)	3.5	4.5 (2.2–9.1)
		Mono-2-ethylhexyl phthalate (MEHP)^b^	0.8	4.0	45.3 (10.9-112.5)	1.2	1.4 (0.6–2.9)
	Di-isononyl phthalate (DiNP)	Mono-isononyl phthalate (MiNP)^a^	0.9	93.3	< LOD	< LOD	0.64 (0.6–1.7)
		Monooxononyl phthalate (MONP)	0.4	21.3	1 (0.5–2.9)	1.8	NA
		Mono carboxyisooctyl phthalate (MCOP)	0.3	10.7	1 (0.3–3.3)	6.9	NA
	Di-isodecyl phthalate (DiDP)	Mono carboxyisononyl phthalate (MCNP)	0.2	0	3.3 (1.5–10.5)	1.7	2.2 (1.1–4.4)
Phthalate replacements	Di(2-ethylhexyl) terephthalate (DEHTP)	Mono-2-ethyl-5-carboxypentyl terephthalate (MECPTP)	0.2	0	20.1 (5.9–59.3)	17.9	NA
		Mono-2-ethyl-5-hydroxyhexyl terephthalate (MEHHTP)	0.4	33.3	1.4 (< LOD-6.3)	4.5	NA
	Di(isononyl) cyclohexane-1,2-dicarboxylate (DINCH)	Cyclohexane-1,2-dicarboxylic acid, mono carboxyisooctyl ester (MCOCH)^a^	0.5	80	< LOD	0.6	NA
		Cyclohexane-1,2-dicarboxylic acid, mono hydroxyisononyl ester (MHNCH)^a^	0.4	64	< LOD (< LOD-0.7)	< LOD	0.3 (0.3–0.9)
Phenols and parabens	Disinfectant related	2,4-Dichlorophenol	0.1	9.3	0.5 (0.2–1.1)	0.5	NA
		2,5-Dichlorophenol	0.1	6.7	1.2 (0.5–6.8)	2.1	NA
		Triclocarban^a^	0.1	52	< LOD (< LOD-0.6)	< LOD	NA
		Triclosan^a^	1.7	52	< LOD (< LOD-4.2)	3.5	4.9 (1.2–24.1)
	Bisphenols	Bisphenol A (BPA)	0.2	4.0	1.0 (0.5-2.0)	1.0	1.3 (0.3–2.6)
		Bisphenol F(BPF)^a^	0.2	56	< LOD (< LOD-0.5)	< LOD	0.3 (0.1–0.9)
		Bisphenol S (BPS)	0.1	24	0.2 (0.1–0.7)	0.5	0.6 (0.2–1.3)
	Personal care product related	Benzophenone-3	0.4	4.0	6.7 (2.4–25.7)	15.5	25.0 (7.5–94.6)
		Methyl paraben^b^	1.0	0	376.7 (68.5-1214.6)	28.8	94 (25.9-316.9)
		Ethyl paraben^b^	1.0	26.7	2.1 (< LOD-16.2)	< LOD	1.9 (0.7–13.9)
		Propyl paraben^b^	0.1	1.3	21.1 (3.9–84.1)	3.2	18.3 (3.1–68.4)
		Butyl paraben^a^	0.1	72	< LOD	< LOD	0.1 (0.1–0.4)

^a^-Excluded in further analysis due to relatively low detection frequencies

^b^- excluded due to relatively high concentrations that are potentially due to the use of medical tubing/medications/wipes at delivery

*Creatinine-corrected urinary biomarker concentrations are measured in micrograms per gram of creatinine (µg/g creatinine)

^+^Biomarker concentrations measured in nanograms per milliliter

LOD-level of detection of biomarkers

### Global metabolome

Maternal (*n* = 72) and cord (*n* = 63) blood serum samples were collected and stored at -80 °C. To conduct metabolome assays, the frozen serum samples were thawed on ice, and 65 µL of each sample was retrieved. Subsequently, 130 µL of acetonitrile, which contained a stable isotope standards mixture, was added to the serum samples. After vortex-mixing, the samples were allowed to equilibrate for 30 min and were then centrifuged to remove proteins. Metabolomic profiling of the serum samples (in 10 µL aliquots) was conducted at Emory University (Atlanta, GA) using the Dionex UltiMate 3000 Ultra-High-Performance Liquid Chromatography (UHPLC) coupled with the Q-Exactive High-Field mass spectrometer from Thermo Scientific. The mobile phase for metabolite separation consisted of formic acid, acetonitrile, and UHPLC-MS grade water. The analysis consisted of triplicate injections, utilizing a hydrophilic interaction liquid chromatographic column for positive electrospray ionization (ESI) and a C-18 column for negative ESI (Walker et al., 2019; Yu et al., 2013). We included distinct columns to maximize the metabolome feature coverage (Soltow et al., 2013).

The mass spectrometer was configured to operate in full scan mode with a resolution of 120,000 over a mass-to-charge (m/z) ratio spanning from 85 to 1,275. The raw data files acquired from the mass spectrometer were processed using the apLCMS R package, enabling the acquisition of m/z ratios, retention times, and feature intensities (Yu et al., 2009). We utilized the ComBat algorithm to address batch effects (*n* = 4) in the metabolic features by adjusting for technical variance in feature intensities arising from different batch runs (Johnson et al., 2007). Feature intensities from triplicate injections were aggregated using the median value using the xMSanalyzer algorithm (Uppal et al., 2017). We excluded features representing metabolites (m/z & retention time combinations) with ≥ 20% non-detectable values or with a coefficient of variation ≥ 100%. This led us to retain 17,064 metabolome features (6,857 in the negative mode and 10,207 in the positive mode) for subsequent analysis. To facilitate downstream analysis, we transformed the feature intensities to the log_10_ scale and standardized them to the Z-score scale (unit scaling).

### Data analysis

#### Metabolome-wide associations (MWAS)

Among the 75 pairs of pregnant participant-infant pairs included in this study, we assessed MWAS between maternal urinary EDC biomarkers at delivery and available metabolome data from 72 maternal serum samples, as well as 63 cord serum samples. We assessed these associations using a multiple linear regression approach, where metabolome feature intensities (17,064 features) were considered as the outcome, and urinary concentrations of EDC biomarkers (adjusted for urine creatinine and log_10_ transformed) were considered as the exposure. The effect estimates from the linear regression were adjusted for factors such as maternal BMI, age, tobacco use, education, and race. Additionally, in the newborn analysis, we adjusted for maternal parity and the newborn’s sex.

Within the regression model, we considered maternal BMI, parity, and age as continuous variables, while tobacco use during pregnancy, education level, race, and newborn sex were considered categorical. Additionally, we considered a threshold of 0.2 (q < 0.2) using the Benjamini-Hochberg (FDR-BH) procedure to determine statistical significance, allowing up to 20% false positives (Benjamini & Hochberg, 1995).

#### Enrichment Analysis

The enrichment analysis was conducted exclusively on the metabolome feature sets when we identified associations between urinary biomarkers and a minimum of 10 metabolome features. In the enrichment analysis, we considered liquid chromatography-mass spectrometry (LC-MS) mixed mode (combining metabolome features from positive and negative modes) and focused on 30 potential adducts for the metabolome features (Table S1). For the quantitative enrichment analysis, we allowed a mass tolerance for up to 5 ppm and 10,000 permutations to putatively annotate metabolites via metabolite set libraries (available through the MetaboAnalystR package) from the input metabolome set. This analysis was performed separately for each urinary EDC biomarker and serum metabolome profile from the participant groups (maternal or newborn) using the mummichog version 2.0 algorithm along with the current version of the Homo Sapiens– MFN pathway library available through the MetaboAnalystR R package version 3.3 (Li et al., 2013b; Pang et al., 2021). We considered features with the 500th smallest raw p-value as a potential threshold. Subsequently, we obtained the pathway names, total serum metabolites, total hits, expected hits, significant hits, and Fisher-exact test outcomes for each pathway. Statistically significant alterations of metabolic pathways were determined using a p-gamma value < 0.05 as a threshold. Enriched pathways were highlighted using the KEGG pathway hierarchies that included 11 groups (Kanehisa & Goto 2000).

## Results

The study comprised pregnant individuals with a median age of 29 years (interquartile range [IQR] 25–32) at delivery, predominantly multiparous (83%), and almost half were non-Hispanic Black (47%) (Table S3). Their median BMI was 26 kg/m² (IQR 23–31), and 17% smoked during pregnancy. Approximately 75% of study participants had less than a 4-year college education, 64% had household incomes below Cincinnati’s median, and all study participants delivered term newborns (range: 37–41 weeks). Among the newborns, 58% were male. The median birth weight of the newborns was 3,269 grams (IQR 3,034 − 3,515), with a median birth length of 50 centimeters (IQR 48–51). The median concentrations of maternal urinary EDC biomarkers, except for mono-2-ethyl-5-carboxypentyl terephthalate (MECPTP), were observed to be lower among our study participants compared to the concentrations among the overall population reported in 2015–2016 NHANES (Table 1). However, the concentrations of Di(2-ethylhexyl) phthalate (DEHP), Di-isodecyl phthalate (DiDP), and personal care product-related phenol metabolites were higher among our study population compared to the pregnant population included in the 2013–2106 NHANES.

**Fig. 1:**
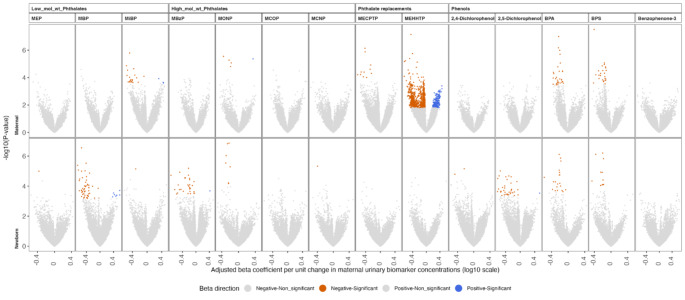
Metabolome-wide associations between maternal urinary biomarkers at delivery and maternal or newborn metabolome. Each panel represents the beta coefficients from the linear regression model between urinary biomarker concentrations and metabolome feature intensities. Metabolome features that did not reach statistical significance (q-value > = 0.2) are coded as gray for both negative and position associations and features having q-value < 0.2 are coded as orange for negative associations and blue for positive associations

We observed associations between six maternal urine biomarkers [bisphenol A (BPA), bisphenol S (BPS), MECPTP, mono-2-ethyl-5-hydroxyhexyl terephthalate (MEHHTP), mono-isobutyl phthalate (MiBP), and monooxononyl phthalate (MONP)] and maternal serum metabolome features (Fig. 1; Table 2). These associations were primarily in the negative direction. Among the maternal urinary biomarkers examined, MEHHTP exhibited a relatively higher number of associations (q-value < 0.2), showing negative associations with 1,114 maternal serum metabolome features. Out of the 14 maternal urinary EDC biomarkers included in this study, 10 biomarkers were associated with the newborn serum metabolome. However, maternal urinary benzophenone-3, monocarboxyisooctyl phthalate (MCOP), MECPTP, and MEHHTP demonstrated null associations with the newborn serum metabolome. Similar to the trends observed in maternal metabolome associations, we noted that newborn metabolome features were predominantly negatively associated with maternal urinary biomarkers. Among the associations in the newborn serum metabolome, mono-n-butyl phthalate (MBP) urinary concentrations displayed the highest number of associations with metabolome features (*n* = 57), the majority of which were negative associations.

**Table 2:** Summary of maternal and newborn metabolome-wide associations with maternal EDC biomarkers

Biomarker	Maternal		Newborn	
	β (+)	β (-)	β (+)	β (-)
MEP	0	0	0	1 (0.006%)
MBP	0	0	7 (0.041%)	50 (0.293%)
MiBP	3 (0.018%)	20 (0.117%)	0	1 (0.006%)
MBzP	0	0	1 (0.006%)	29 (0.170%)
MONP	1 (0.006%)	4 (0.023%)	0	7 (0.041%)
MCOP	0	0	0	0
MCNP	0	0	0	1 (0.006%)
MECPTP	0	11 (0.064%)	0	0
MEHHTP	192 (1.125%)	1114 (6.528%)	0	0
2,4-dichlorophenol	0	0	0	2 (0.012%)
2,5-dichlorophenol	0	0	1 (0.006%)	35 (0.205%)
Benzophenone-3	0	0	0	0
BPA	0	29 (0.170%)	0	20 (0.117%)
BPS	0	25 (0.147%)	0	11 (0.064%)

This table contains the frequency and percent of metabolome features associated with urinary EDC biomarkers using q-value < 0.2 as a threshold for statistical significance. Beta-positive/negative represents the frequency and percentage of metabolome features associated with maternal biomarkers. EDC exposure biomarkers were measured in maternal urine at delivery visit. Global metabolome was measured from maternal and cord serum

**Fig. 2:**
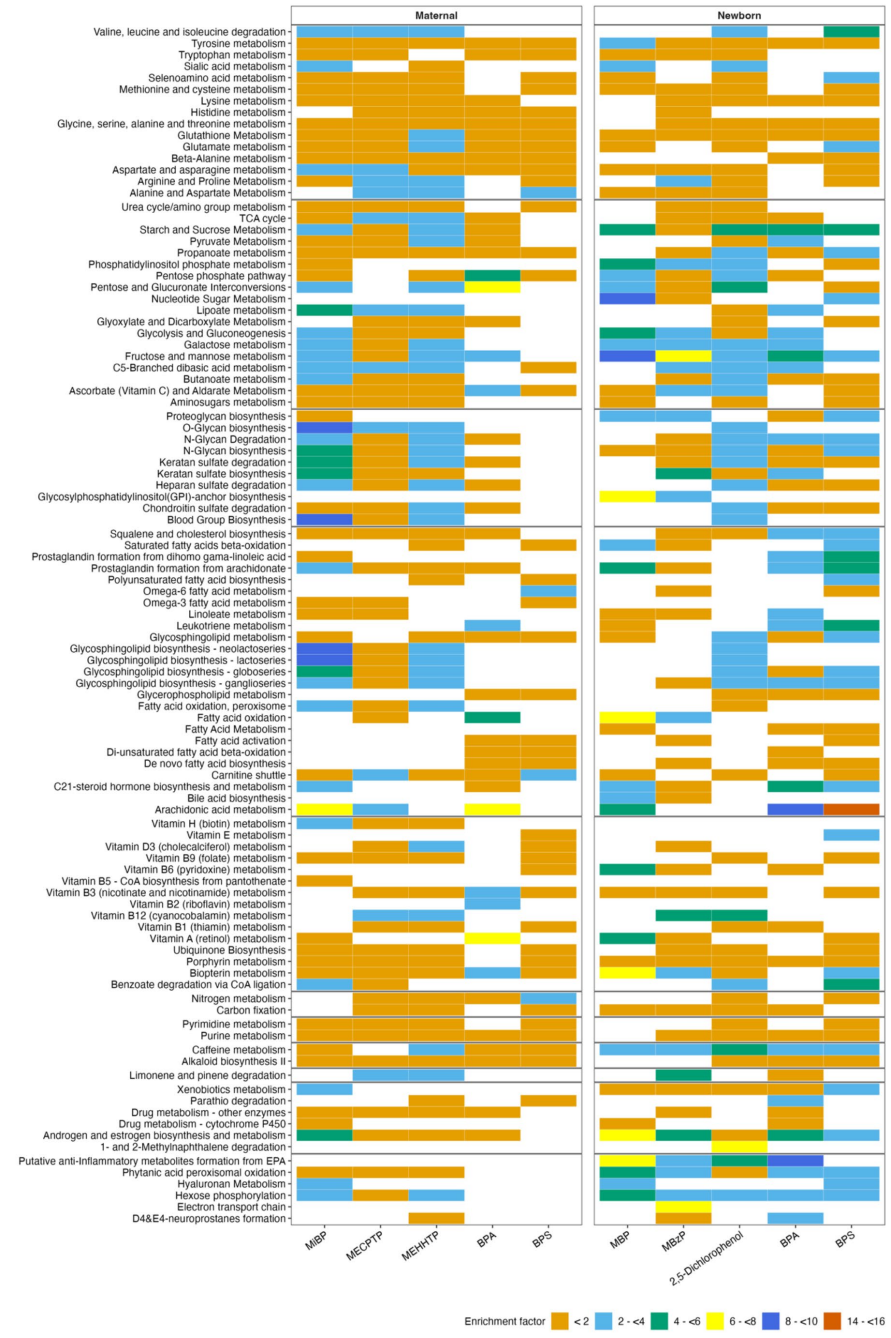
Pathway enriched (p-gamma < 0.05) using 500 metabolome features with the lowest raw p-values. Highlighted urinary biomarkers with at least 10 metabolome features associated with biomarker concentrations using q-value < 0.2. Enrichment factor is the ratio between the number of statistically significant hits and the number of expected hits per pathway

Based on the MWAS results, we constrained our enrichment analysis to focus on five EDC biomarkers (BPA, BPS, MiBP, MECPTP, and MEHHTP) in pregnant individuals and five EDC biomarkers [2,5-dichlorophenol, BPA, BPS, MBP, monobenzyl phthalate (MBzP)] in newborns with at least 10 serum metabolome features associated with maternal urinary biomarkers. Due to a relatively lower number of metabolome features associated with maternal urinary biomarkers that led to convergence issues, we performed the enrichment analysis by considering 500 features with the lowest p-values from the MWAS results (p-value thresholds available in Table S2). From the pathway analysis, we observed that maternal urinary MEHHTP and MiBP concentrations were associated with the highest number (*n* = 71 pathways) of metabolic pathways from the maternal serum metabolome (Fig. 2). Among these pathways associated with the maternal metabolome, the majority (16 pathways) are related to carbohydrate metabolism. Of these carbohydrate metabolism pathways, the lipoate metabolism pathway was enriched at a relatively higher degree for MiBP, and the TCA cycle, starch/sucrose, pyruvate, pentose, lipoate, galactose, fructose, mannose, C5 branched dibasic acid metabolism pathways were enriched at a higher degree for MEHHTP. Furthermore, similar pathways were enriched in both the maternal and newborn serum metabolome while using 500 features with relatively smallest p-values corresponding to all the urinary EDC biomarkers included for MWAS in this study (Figure S1).

In newborns, we noted that maternal urinary 2,5-dichlorophenol concentrations were associated with a relatively higher number (*n* = 67 pathways) of metabolic pathways. Among these 67 altered pathways in newborns, the majority can be categorized as carbohydrate (*n* = 17) and amino acid metabolism pathways (*n* = 13) (Figure S2). Specifically, pentose/glucuronate interconversions and starch/sucrose metabolism pathways from the carbohydrates group were enriched at a higher magnitude.

Among the metabolic pathways, we observed 37 metabolism pathways commonly associated between 2,5-dichlorophenol and BPA with maternal metabolome (Figure S3). Among these shared pathways, the predominant categories included carbohydrate (*n* = 11) and glycan metabolism (*n* = 6). Furthermore, maternal urinary concentrations of MiBP and MEHHTP were commonly associated with 57 metabolic pathways; while using maternal metabolome, these pathways were predominantly related to carbohydrate (*n* = 15) and amino acid metabolism (*n* = 12). Additionally, maternal urinary BPA concentrations were commonly associated with 29 pathways considering maternal and newborn metabolome with predominantly lipid metabolism pathways (*n* = 9). Moreover, BPS concentrations were commonly associated with 30 metabolic pathways while considering maternal and newborn metabolome, with the majority (*n* = 9) overlapping in amino acid metabolism.

## Discussion

In this study, we observed associations between maternal urinary biomarker concentrations of phenols and phthalates at delivery and maternal/newborn serum metabolome. The enrichment of these metabolome features in maternal and newborn serum indicated alterations in the metabolism of various components, including amino acids, carbohydrates, glycan, lipids, vitamins, and other cofactors. Alterations of these metabolic pathways may influence fetal growth (as a result of altered metabolism of amino acids, carbohydrates, vitamins, and cofactors), fetal neurodevelopment (lipids), and alterations in protein function/signaling molecules (glycans) (Li et al., 2022; Padmanabhan et al., 2021; Varki, 2017). The alterations of metabolic pathways were primarily driven by urinary concentrations of phthalate metabolites (MiBP, MBP, MBzP), phthalate replacements (MECPTP, MEHHTP), and phenols (2,5-dichlorophenol, BPA, BPS).

EDCs are known to interact with PPAR, steroid, and thyroid receptors, which play a role in transcription factors involved in fetal development, developmental disorders, metabolism, and homeostasis (Gronemeyer et al., 2004; Kim & Park, 2014). Previous observational studies have highlighted the influence of endocrine-disrupting chemicals on metabolic reprogramming and its potential impact on adiposity, metabolism, and cardiovascular outcomes in later life (Ghassabian et al., 2022; Kiess et al., 2021; Radke et al., 2019). In this study, we focused on exploring maternal and newborn metabolomic changes associated with exposures to non-persistent EDCs at delivery (assuming recurrent exposures).

Our findings align with recent studies that explored third-trimester exposure to phthalates and revealed alterations in placenta metabolome, indicating altered carbohydrate (glucitol and n-acetylneuraminate) and lipid (carnitine, O-acetylcarnitine, and 2-hydroxybutyrate) metabolism (Parenti et al., 2022). Additionally, a study based on a rat model suggested associations between exposure biomarkers of chemicals often detected in personal care products (diethyl phthalate, methyl paraben, and triclosan) and alterations in carbohydrate (pyruvate) and lipid (fatty acid) metabolism (Houten et al., 2016). However, a large cohort study involving 1,074 children reported null associations between gestational urinary phthalate metabolite concentrations and metabolome in cord blood or at age 1 year (Thomson et al., 2023). Exposure to EDCs during pregnancy may not limit their impact on fetal health. Few studies have highlighted associations between phthalate replacement (MECPTP) concentrations during pregnancy and maternal outcomes, such as long-term maternal weight and glycemia biomarkers (Deierlein et al., 2022; Wu et al., 2021). However, the underlying alterations of metabolism pathways are unclear.

Studies based on animal models (zebrafish and rats) exposed to bisphenols (BPA and BPS) demonstrated altered amino acid, nucleotide, and carbohydrate metabolism (Mao et al., 2021; Tremblay-Franco et al., 2015; Yoon et al., 2017). Another study involving zebrafish suggested associations between benzophenone-3 and altered vitamin and cofactor metabolism (Wang et al., 2023). Additional studies on pregnant mice indicated that prenatal concentrations of benzophenone-3 were associated with altered carbohydrate (fructose, mannose, citrate cycle) and amino acid (arginine and proline) metabolism in the fetus (Han et al., 2022).

Overall, our findings suggest associations between exposure to EDCs at delivery and potential alteration of amino acids, lipids, glycan, carbohydrates, vitamins, and cofactors, consistent with existing preclinical or human-based studies (Houten et al., 2016; Mao et al., 2021; Parenti et al., 2022; Tremblay-Franco et al., 2015; Wang et al., 2023; Yoon et al., 2017). Our findings support potential underlying mechanisms underpinning the associations between non-persistent EDC exposures and cardiometabolic conditions reported in the literature (Ghassabian et al., 2022; Kiess et al., 2021; Radke et al., 2019). In contrast to Thomson et al., we excluded the DEHP metabolites because their concentrations could be attributed to medical procedures during delivery visits (Vandentorren et al., 2011; Yan et al., 2009). However, both human based studies (Parenti et al. and Thomson et al.) considered associations between gestational phthalates and newborn metabolome were limited with the coverage of the metabolites measured from the serum. Parenti et al. included 54 placenta samples and quantified concentrations of 54 metabolites using Chemnox profiler (V 8.1) and confirmed using internal reference (DSS-d6) standards (Parenti et al., 2022). Thomson et al. quantified 17 metabolites from cord blood and child age at year 1 using internal standards that are related to the central carbon metabolism (Thomson et al., 2023).

Both studies, conducted by Parenti et al. and Thomson et al., utilized proton NMR spectroscopy to quantify low molecular weight metabolites from the metabolome profile. Both of these studies measured metabolome features using internal standards that enhance the confidence of metabolite annotations. However, this limits metabolite coverage, potentially leading to an underrepresentation of metabolomic alterations associated with exposures. In our study, we adopted an untargeted approach using LC-MS that yields global coverage of metabolome features. However, it is essential to acknowledge potential biases introduced by the putative identification of metabolome features during the processing of MS2 data for pathway enrichment analysis using the mummichog algorithm (Li et al., 2013a). Given the heterogeneity in annotating metabolome features across scientific facilities, future studies supplementing untargeted metabolomics data with metabolome features annotated using internal standards may enhance the validity of findings (Yu et al., 2019). Additionally, further longitudinal studies that consider serum metabolites annotated from the untargeted metabolome as potential mediators when exploring associations between chemical biomarkers and phenotypes would strengthen the robustness of our findings. Our findings are based on the convenient sampling of study participants recruited from a US metropolitan area and may not represent the general US pregnant population, which limits generalizability. Furthermore, since diet could influence metabolome profile (especially amino acids and xenobiotics), future studies using fasting serum samples are needed to validate current findings (Agueusop et al., 2020; Bar et al., 2020). Data on in-vitro fertilization treatment or hormonal therapies/contraceptives were not available to account for their impact on the metabolome.

## Conclusion

We observed metabolic alterations in maternal/newborn serum profiles associated with exposure to non-persistent EDCs at delivery, including certain phthalates and phenol urinary biomarkers. Notably, biomarkers of phenols (BPA, BPS, 2,5-dichlorophenol), phthalates (MBP, MiBP, MBzP), and phthalate replacements (MECPTP and MEHHTP) played a key role in these associations. While we employed an untargeted metabolomics approach for comprehensive serum metabolome coverage, acknowledging uncertainties in putative identification using the mummichog algorithm, future studies supplementing global metabolomic data with feature annotations using reference standards for level-1 confidence could enhance validity. Additionally, considering prospective repeated exposure/metabolome measurements and follow-up on newborn phenotypes in further studies may establish exposure-outcome associations mediated by metabolome alterations during critical periods such as pregnancy.

## Electronic supplementary material

Below is the link to the electronic supplementary material.

Supplementary Material 1

## Data Availability

The datasets generated and/or analyzed during the current study are not publicly available due to our extended ongoing analysis.
