# Supplementary material for: Global metabolomic alterations associated with endocrine-disrupting chemicals among pregnant individuals and newborns
Source: Metabolomics. 2025 Jan 25;21(1):20. doi: 10.1007/s11306-024-02219-7 (PMC11762426; doi:10.1007/s11306-024-02219-7)
Supplement: Supplementary file 1 — Supplementary Material 1 [file 11306_2024_2219_MOESM1_ESM.docx]

Supplemental information: Global metabolomic alterations associated with endocrine-disrupting chemicals among pregnant individuals and newborns

Jagadeesh Puvvula*, Lucie C. Song, Klaudia J. Zalewska, Ariel Alexander, Kathrine E. Manz, Joseph M. Braun, Kurt D. Pennell, Emily A. DeFranco, Shuk-Mei Ho, Yuet-Kin Leung, Shouxiong Huang, Ann M. Vuong, Stephani S. Kim, Zana Percy, Priyanka Bhashyam, Raymund Lee, Dean P. Jones, Vilinh Tran, Dasom V. Kim, Antonia M. Calafat, Julianne C. Botelho, Aimin Chen

*Corresponding author:

Jagadeesh Puvvula - Department of Biostatistics, Epidemiology and Informatics, Perelman School of Medicine, University of Pennsylvania, Philadelphia, PA; email: [Jagadeesh.Puvvula@pennmedicine.upenn.edu](mailto:Jagadeesh.Puvvula@pennmedicine.upenn.edu)

Contents (7 pages): 3 tables & 3 figures

[Table S1. Metabolite adducts 2](#_Toc172619819)

[Table S2. Raw p-value thresholds for enrichment analysis 2](#_Toc172619820)

[Table S3. Population characteristics (n=72) 3](#_Toc172619821)

[Figure S1.Pathway enrichment using 500 metabolome features with the smallest p-values. This figure includes enrichment results for all the urinary biomarkers included for MWAS in this study. 5](#_Toc161391301)

[Figure S2.Metabolic pathways enriched (p-gamma <0.05) for select chemical biomarkers by KEGG pathway groups. This figure excludes pathways that are not included in the KEGG hierarchy. 6](#_Toc161391302)

[Figure S3. Metabolic pathways enriched (p-gamma <0.05) and overlapped for select chemical biomarkers and metabolome sets. 7](#_Toc161391303)

| Table S1. Metabolite adducts | |
| --- | --- |
| List of metabolite adducts (n=30) | M+FA-H [1-], M-H [1-], 2M-H [1-], M-H+O [1-], M(C13)-H [1-], 2M+FA-H [1-], M-3H [3-], M-2H [2-], M+ACN-H [1-], M+HCOO [1-], M+CH3COO [1-], M-H2O-H [1-], M [1+], M+H [1+], M+2H [2+], M+3H [3+], M+H2O+H [1+], M-H2O+H [1+], M(C13)+H [1+], M(C13)+2H [2+], M(C13)+3H [3+], M-NH3+H [1+], M+ACN+H [1+], M+ACN+2H [2+], M+2ACN+2H [2+], M+3ACN+2H [2+], M+NH4 [1+], M+H+NH4 [2+], 2M+H [1+], 2M+ACN+H [1+] |
| Above mentioned potential metabolite adduct forms were considered while performing enrichment analysis to putatively annotate metabolites. | |

| Table S2. Raw p-value thresholds for enrichment analysis | | |
| --- | --- | --- |
| Biomarker | Maternal | Newborn |
| MEP | 0.0652 | 0.0354 |
| MBP | 0.0350 | 0.0141 |
| MiBP | 0.0101 | 0.0206 |
| MBzP | 0.0383 | 0.0089 |
| MONP | 0.0457 | 0.0434 |
| MCOP | 0.0348 | 0.0295 |
| MCNP | 0.0239 | 0.0394 |
| MECPTP | 0.0095 | 0.0495 |
| MEHHTP | 0.0050 | 0.0477 |
| 2,4-dichlorophenol | 0.0769 | 0.0137 |
| 2,5-dichlorophenol | 0.0432 | 0.0106 |
| Benzophenone-3 | 0.0466 | 0.0317 |
| BPA | 0.0186 | 0.0385 |
| BPS | 0.0252 | 0.0375 |
| Raw p-value thresholds are the 500^th^ smallest p-values obtained from the MWAS results. | | |

| Table S3. Population characteristics (n=72) | |
| --- | --- |
| Variable | Value |
| Maternal age (years)^a^ | 29 (24.8-32.0) |
| Gestational age (weeks)^a^ | 39 (38.7-39.0) |
| Maternal BMI^a^ | 25.9 (22.5-30.5) |
| Parity^b^ | 1: 12 (16.7)  2: 34 (47.2)  3: 9 (12.5)  ≥4: 17 (23.6) |
| Smoking during pregnancy^b^ | Yes: 12 (16.7)  No: 60 (83.3) |
| Education^b^ | Less than a bachelor’s degree: 54 (75.0)  Greater or equal to a bachelor’s degree: 18 (25.0) |
| Race^b^ | Non-Hispanic White: 29 (40.3)  Non-Hispanic Black: 34 (47.2)  Hispanic: 6 (8.3)  Other: 3 (4.2) |
| Household income^b^ | Below median for Cincinnati: 46 (64)  Above median for Cincinnati: 26 (36) |
| Newborn birth weight (grams)^a^ | 3269 (3034-3515) |
| Newborn birth length (Centimeters)^a^ | 50 (48-51) |
| Newborn sex^b^ | Male: 42(58.3)  Female: 30(41.7) |
| a-median and interquartile range & b-count and percentage | |


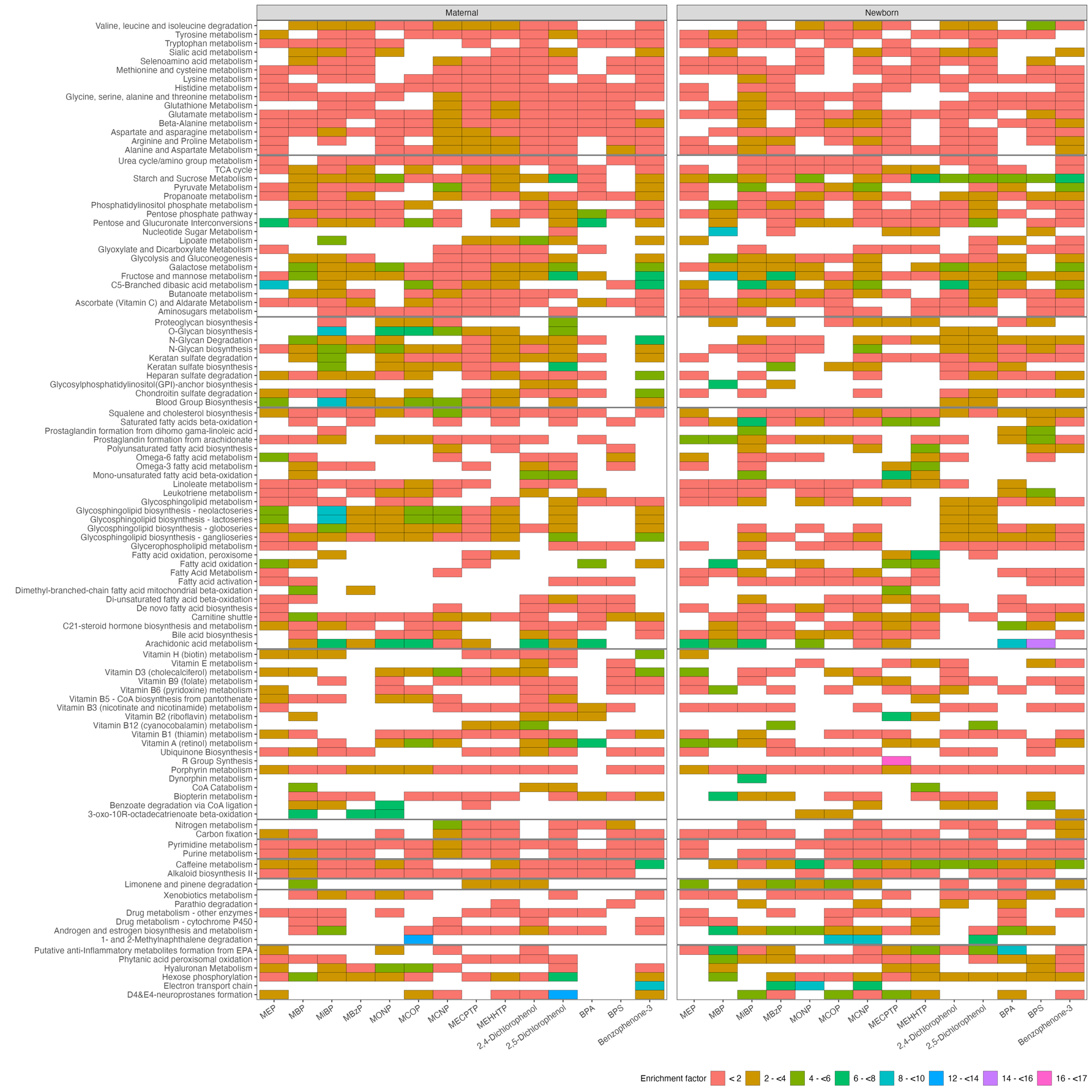


Figure S1.Pathway enrichment using 500 metabolome features with the smallest p-values. This figure includes enrichment results for all the urinary biomarkers included for MWAS in this study.

Figure S2.Metabolic pathways enriched (p-gamma <0.05) for select chemical biomarkers by KEGG pathway groups. This figure excludes pathways that are not included in the KEGG hierarchy.Figure S1.Pathway enrichment using 500 metabolome features with the smallest p-values. This figure includes enrichment results for all the urinary biomarkers included for MWAS in this study.

Figure S2.Metabolic pathways enriched (p-gamma <0.05) for select chemical biomarkers by KEGG pathway groups. This figure excludes pathways that are not included in the KEGG hierarchy.

Figure S3. Metabolic pathways enriched (p-gamma <0.05) and overlapped for select chemical biomarkers and metabolome sets.Figure S2.Metabolic pathways enriched (p-gamma <0.05) for select chemical biomarkers by KEGG pathway groups. This figure excludes pathways that are not included in the KEGG hierarchy.


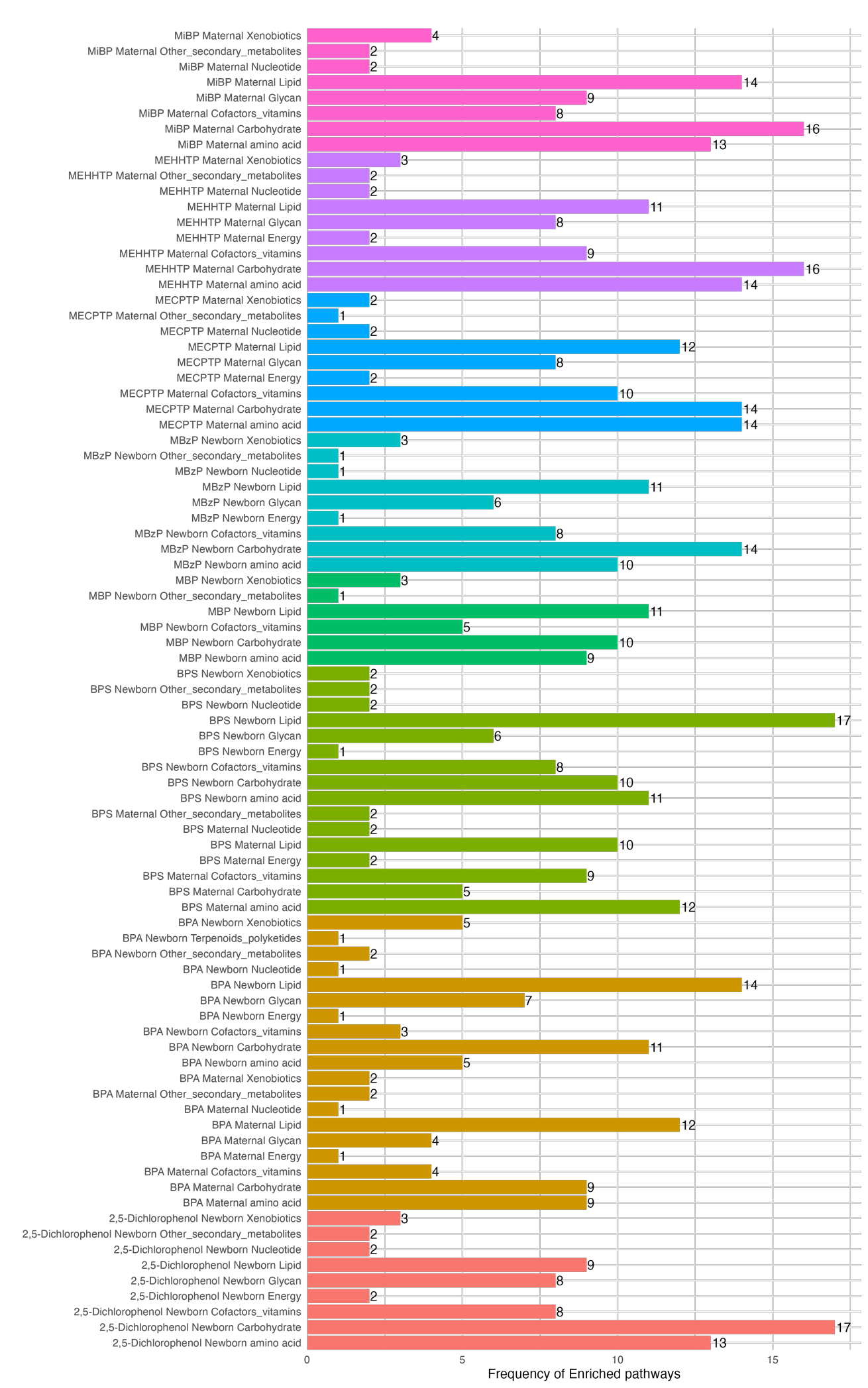

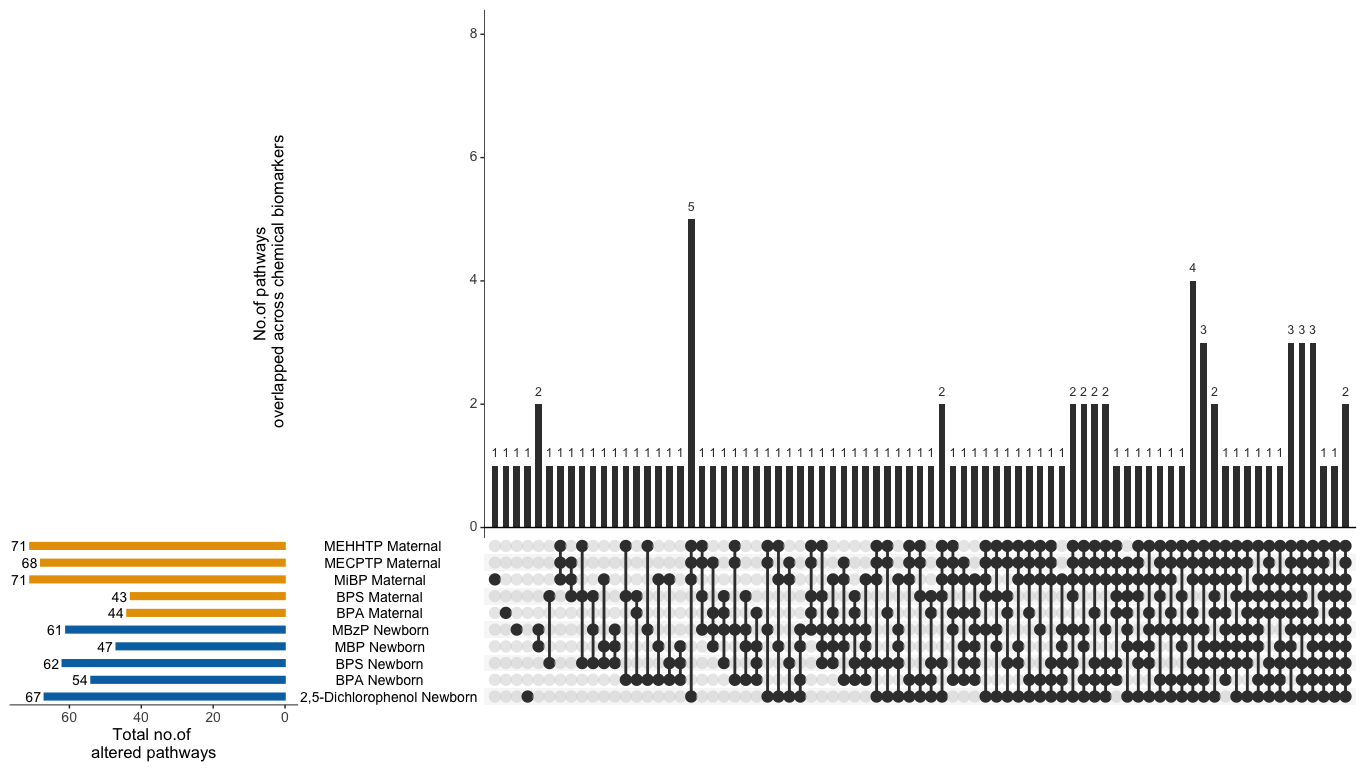


Figure S3. Metabolic pathways enriched (p-gamma <0.05) and overlapped for select chemical biomarkers and metabolome sets.
